# Supplementary material for: Sound generation in zebrafish with Bio-Opto-Acoustics
Source: Nat Commun. 2020 Nov 30;11:6120. doi: 10.1038/s41467-020-19982-5 (PMC7705743; doi:10.1038/s41467-020-19982-5)
Supplement: Supplementary file 1 — Supplementary Information [file 41467_2020_19982_MOESM1_ESM.pdf]

Sound generation in zebrafish with Bio-Opto-Acoustics

Supplementary Information

Itia A. Favre-Bulle, Michael A. Taylor, Emmanuel Marquez-Legorreta, Gilles Vanwalleghem,

Rebecca E. Poulsen, Halina Rubinsztein-Dunlop, Ethan K. Scott

Modelling of Otolith motion under periodic stimulation

The motion of each otolith undergoing sound pressure or BOA stimulation can be studied analytically using Newton's law:

$$\sum \vec{F} = m\vec{a} \tag{1}$$

The forces present in the direction of the BOA stimulation when the otolith is manipulated are the viscous drag from the ear fluid F_D, the pulling forces of the hair cells F_{HC} on the otolith and a force F which can include optical force, acoustic sounds, and background forces such as heartbeat, blood flow, muscle movement, and thermal fluctuations.

$$F(t) + F_D(t) + F_{HC}(t) = ma \tag{2}$$

Under the assumption that the hair cells are elastic, and that we apply optical forces and sound pressure in the elastic regime of hair cells, we can deduce from Hooke's law that F_{HC} can be expressed as $F_{HC} = -kx$, where k is the spring constant and x is the otolith displacement. Therefore:

$$m\frac{d^2x}{dt^2} + \gamma\frac{dx}{dt} + k\,x(t) = F(t) \tag{3}$$

Where γ is the viscous drag coefficient, and m is the mass of the otolith. This second order differential equation can be solved in the Fourier domain, which would give us[1]:

$$x(f) = \frac{F(f)}{k + i\,2\,\pi\gamma f - m(2\,\pi\,f)^2} \tag{4}$$

At low frequencies we have $x{\sim}F/k$. The displacement amplitude is limited by the hair cell stiffness. At higher frequency we anticipate viscous drag to dominate, which causes the oscillation amplitude to scale as 1/f. Inertial motion becomes significant at high frequency ($f > \gamma/m$), but the damping is expected to be sufficiently large that inertial motion is not significant at auditory frequencies. Therefore, we can approximate the otolith displacement x in the Fourier domain to be:

$$x(f) = \frac{F(f)}{k + i\,2\,\pi\gamma f} \tag{4}$$

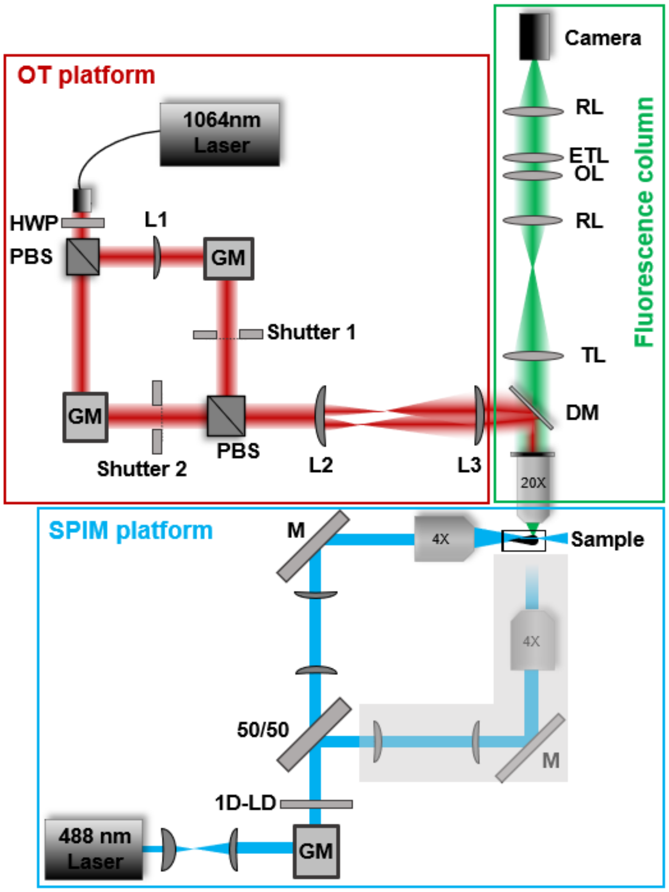

Supplemental Figure 1. Optical setup details. The optical setup combines three optical systems: Dual optical tweezers (red box), light sheet illumination (blue box) and fluorescence imaging (green box). See details for the roles of each optical elements in the Methods. The greyed area on the light sheet platform represents the beam coming from the side of the fish, which is flattened here to produce a 2D image. In fact, this beam's path would be out of the plane of the image (represented in 3D in Fig 1a). HWP, half wave plate; PBS, polarizing beam splitter; L, lens; M, galvo mirrors; DM, dichroic mirror; TL, tube lens; RL, relay lens; OL, offset lens; ETL, electrical tunable lens; M, mirror; 50/50, 50/50 splitter; 1D-LD; one dimension line diffuser.

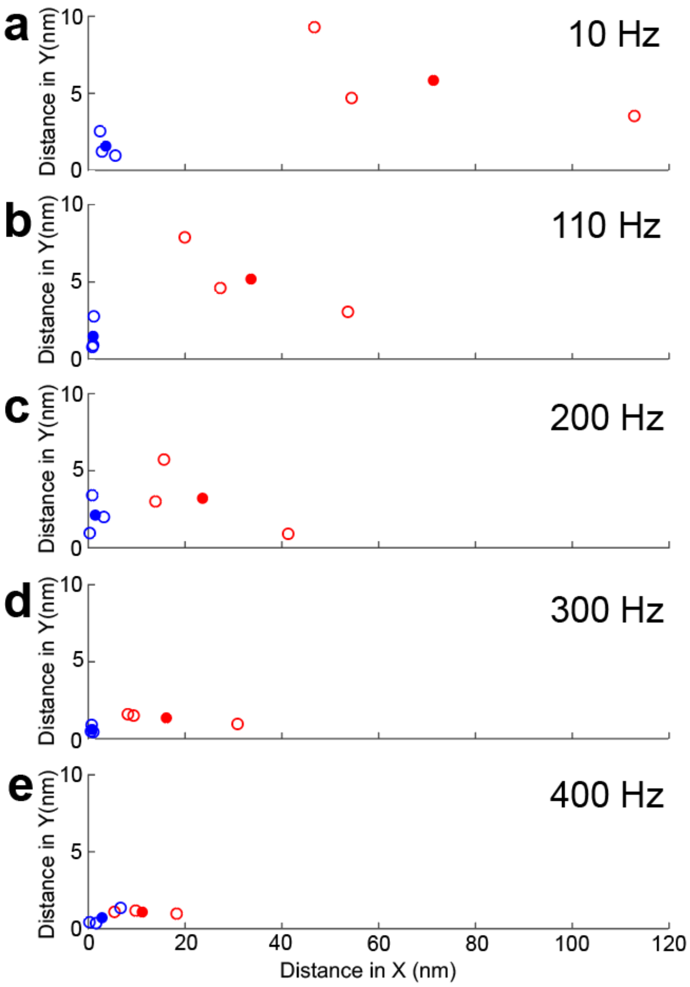

Supplemental Figure 2. BOA stimulation does not appreciably move the off-target otolith. a-e. Measurement of displacements of utricle (red) and saccule (blue) otoliths in X and Y directions (same fish, same ear, same stimulus train, different times) when the utricular otolith undergoes BOA stimulation in the X direction. Hollow circles represent data points for each fish. Filled circles represent the mean value across fish. N=3 fish.

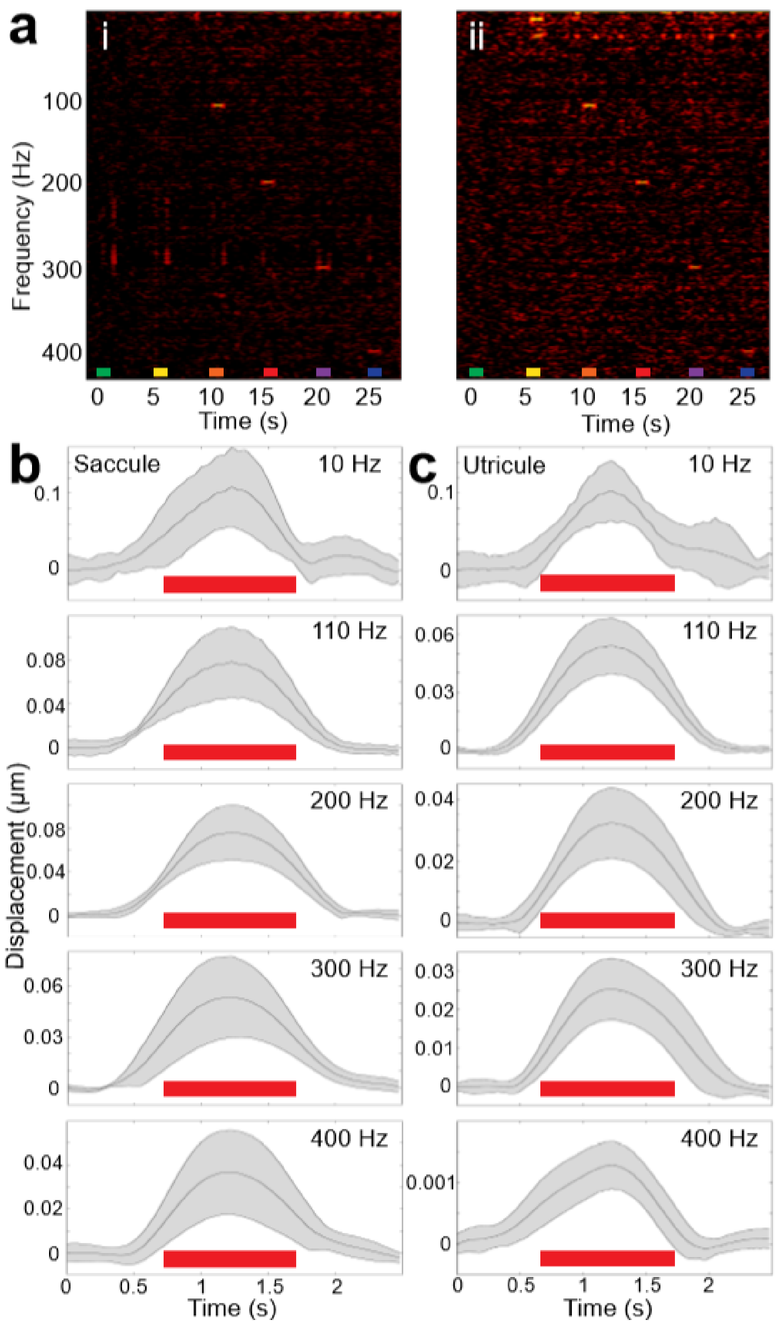

Supplemental Figure 3. Detecting otolith movements at specific frequencies under optical trapping. a. Spectrograms of the displacement of one otolith with a 1s window. Colored boxes represent the time at which each stimulus is presented, the color represents the frequency. Green 1Hz, yellow 10Hz, orange 110Hz, red 200Hz, purple 300Hz and blue 400Hz. **a(i).** Example spectrogram of displacement in the BOA stimulation direction of one saccular otolith in one fish. **a(ii).** Example spectrogram of displacement in the BOA stimulation direction of one utricular otolith in one fish. **b., c**. Average curves (solid line) and standard deviation (grey area) across 5 fish of the amplitude envelopes at specific frequencies (10, 110, 200, 300 and 400) detected for movements of the saccular (b), and utricular (c) otoliths under optical manipulation. Each curve corresponds to one line of the spectrogram, truncated around the time of the stimulus. Red bar shows the time of BOA stimulation (1s in length). The apparent rise in movement prior to stimulus onset is a product of temporal averaging (See Methods).

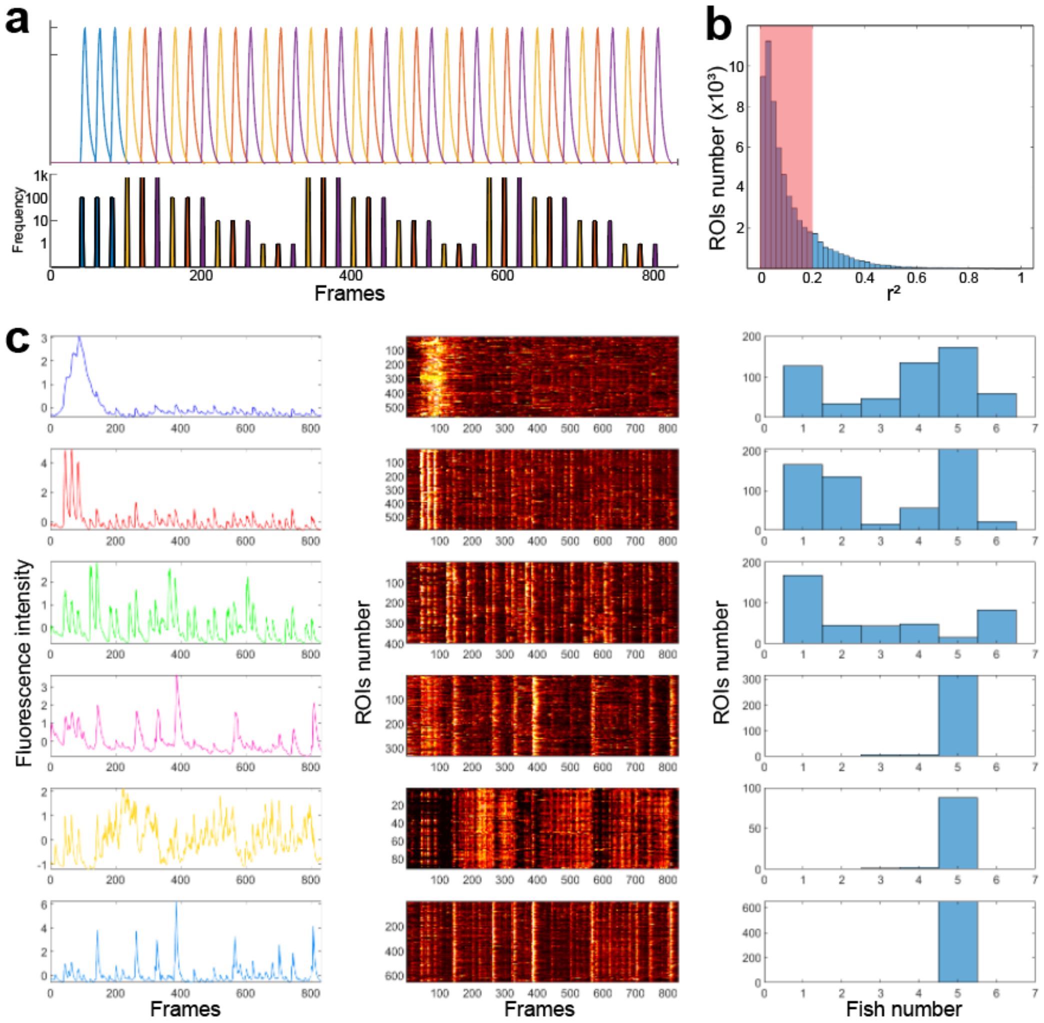

Supplemental Figure 4. Filtering of clusters. a. Top line: regressors used for the linear regression step. Blue corresponds to the auditory tone, yellow to trapping of the utricular otolith, red to trapping of the saccular otolith, and purple to simultaneous trapping of both otoliths. Bottom line: stimulus train at various frequencies, with colors coded as above. **b.** Histogram of r^2 values showing the 0.2 threshold applied to all ROIs after the linear regression step. **c**. All clusters with average responses to tones > 1SD. The first column shows the average traces of all ROIs in the cluster, and the second column shows raster plots of the individual ROIs' responses. Third column shows the number of ROIs drawn from each of the seven fish in the dataset. The first, fourth, fifth, and sixth clusters failed to meet the exclusion criteria (see Methods) and were not analyzed further. Cluster 1 did not pass selection criterion 1, while clusters 4, 5 and 6 did not pass selection criterion 3.

References

1. Berg-Sørensen, K., and Flyvbjerg, H. (2004). Power spectrum analysis for optical tweezers. Review of Scientific Instruments *75*, 594-612.
