# Supplementary material for: Sound generation in zebrafish with Bio-Opto-Acoustics
Source: Nat Commun. 2020 Nov 30;11:6120. doi: 10.1038/s41467-020-19982-5 (PMC7705743; doi:10.1038/s41467-020-19982-5)
Supplement: Supplementary file 2 — Reporting Summary [file 41467_2020_19982_MOESM2_ESM.pdf]

nature research

Corresponding author(s): Itia Favre-Bulle
Ethan Scott

Last updated by author(s): Aug 19, 2020

Reporting Summary

Nature Research wishes to improve the reproducibility of the work that we publish. This form provides structure for consistency and transparency in reporting. For further information on Nature Research policies, see our Editorial Policies and the Editorial Policy Checklist.

Statistics

For all statistical analyses, confirm that the following items are present in the figure legend, table legend, main text, or Methods section.

n/a	Confirmed	
☐	☒	The exact sample size (*n*) for each experimental group/condition, given as a discrete number and unit of measurement
☐	☒	A statement on whether measurements were taken from distinct samples or whether the same sample was measured repeatedly
☒	☐	The statistical test(s) used AND whether they are one- or two-sided<br>*Only common tests should be described solely by name; describe more complex techniques in the Methods section.*
☒	☐	A description of all covariates tested
☐	☒	A description of any assumptions or corrections, such as tests of normality and adjustment for multiple comparisons
☐	☒	A full description of the statistical parameters including central tendency (e.g. means) or other basic estimates (e.g. regression coefficient) AND variation (e.g. standard deviation) or associated estimates of uncertainty (e.g. confidence intervals)
☒	☐	For null hypothesis testing, the test statistic (e.g. *F*, *t*, *r*) with confidence intervals, effect sizes, degrees of freedom and *P* value noted<br>*Give P values as exact values whenever suitable.*
☒	☐	For Bayesian analysis, information on the choice of priors and Markov chain Monte Carlo settings
☒	☐	For hierarchical and complex designs, identification of the appropriate level for tests and full reporting of outcomes
☒	☐	Estimates of effect sizes (e.g. Cohen's *d*, Pearson's *r*), indicating how they were calculated

Our web collection on statistics for biologists contains articles on many of the points above.

Software and code

Policy information about availability of computer code

Data collection	Data collection was performed via uManager 1.4.22. A custom code was written in Java 1.8.0_172 (64bit) in uManager 1.4.22. This code was driving all optical devices (galvo mirrors, laser, camera) and recording all data from the camera.<br>Data and Codes are available here: https://doi.org/10.14264/9809ff7
Data analysis	The volumetric scan (data from camera) was first transformed into a hyperstack with a custom code in Fiji (package manager of uManager 1.4.22). We used the CaImAn package version 1.0 to analyze our images and extract the fluorescent traces of each ROI from every slice (http://github.com/flatironinstitute/CaImAn). The greedy roi method was used to initialize 4000 components from which to extract, demix, and denoise the fluorescent traces using an autoregressive model of order 1. We used a correlation threshold of 0.8 to merge overlapping ROIs and avoid over-segmentation. The components were updated before and after the merge steps, empty components were discarded, and the components were ranked for fitness as in. We used Advanced Normalization Tools (ANTs, https://github.com/ANTsX/ANTs) to compute the diffeomorphic map between the time-averaged 3D image stack of each fish and the H2B-RFP reference of Z-brain. The same mapping was used to warp the centroid coordinates for each ROI of interest to the H2B-RFP frame of reference, which includes 294 segmented brain regions. The active ROIs and their respective fluorescent traces were further analyzed in MATLAB R2018b with a custom-written code. We used a custom code on MATLAB R2018b to represent each ROI centroid as a sphere within the Zbrain reference brain image.<br>Data and Codes are available here: https://doi.org/10.14264/9809ff7

For manuscripts utilizing custom algorithms or software that are central to the research but not yet described in published literature, software must be made available to editors and reviewers. We strongly encourage code deposition in a community repository (e.g. GitHub). See the Nature Research guidelines for submitting code & software for further information.

Data

Policy information about availability of data

All manuscripts must include a data availability statement. This statement should provide the following information, where applicable:

- Accession codes, unique identifiers, or web links for publicly available datasets
- A list of figures that have associated raw data
- A description of any restrictions on data availability

The data and codes that support the findings of this study are available here: https://doi.org/10.14264/9809ff7

Field-specific reporting

Please select the one below that is the best fit for your research. If you are not sure, read the appropriate sections before making your selection.

- [x] Life sciences
- [ ] Behavioural & social sciences
- [ ] Ecological, evolutionary & environmental sciences

For a reference copy of the document with all sections, see nature.com/documents/nr-reporting-summary-flat.pdf

Life sciences study design

All studies must disclose on these points even when the disclosure is negative.

Sample size	n=5 fish for the mechanics of sound perception study (figure 2),<br>n=6 fish for the response distribution study (figure 3).
Data exclusions	For the mechanics of sound perception study (figure 2), videos with the best contrast were kept.<br>For the response distribution study (figure 3), clusters were filtered with the following selection criteria:<br>1. Responsivity to each tone stimuli as a GCaMP6s profile,<br>2. Responsivity to each tone stimuli with a response above 1SD to base line,<br>3. Less than 90% of the ROIs within the cluster are represented in a single fish.
Replication	Each experiment was repeated independently for each of the 5 or 6 fish. Animals from different clutches are used and had similar results. two animals per clutch, 3 clutches in total.
Randomization	As we do not perform treatments or compared groups of animals, no randomization is necessary
Blinding	As we do not perform treatments or compared groups of animals, no blinding is necessary

Reporting for specific materials, systems and methods

We require information from authors about some types of materials, experimental systems and methods used in many studies. Here, indicate whether each material, system or method listed is relevant to your study. If you are not sure if a list item applies to your research, read the appropriate section before selecting a response.

Materials & experimental systems

n/a	Involved in the study	
x		Antibodies
x		Eukaryotic cell lines
x		Palaeontology and archaeology
	x	Animals and other organisms
x		Human research participants
x		Clinical data
x		Dual use research of concern

Methods

n/a	Involved in the study	
x		ChIP-seq
x		Flow cytometry
x		MRI-based neuroimaging

Animals and other organisms

Policy information about studies involving animals; ARRIVE guidelines recommended for reporting animal research

Laboratory animals	Zebrafish (Danio rerio) larvae, 6 days post fertilized, of either sex, were used for the experiments. All experiments were carried out in nacre mutant elavl3:H2B-GCaMP6s larvae of the TL strain.
Wild animals	The study did not involve wild animals.

Field-collected samples	The study did not involve field-collected sample.
Ethics oversight	All procedures were performed with approval from The University of Queensland Animal Welfare Unit (in accordance with approval SBMS/378/16)

Note that full information on the approval of the study protocol must also be provided in the manuscript.
